# Artificial intelligence and ChatGPT in Orthopaedics and sports medicine

**DOI:** 10.1186/s40634-023-00642-8

**Published:** 2023-07-26

**Authors:** Aly M. Fayed, Nacime Salomao Barbachan Mansur, Kepler Alencar de Carvalho, Andrew Behrens, Pieter D’Hooghe, Cesar de Cesar Netto

**Affiliations:** 1 Department of Orthopaedics and Rehabilitation, University of Iowa Hospitals and Clinics, Iowa City, IA USA; 2 Aspetar Orthopedic and Sports Medicine Hospital, Doha, Qatar; 3 Department of Orthopaedic Surgery, Duke University, Durham, NC USA

**Keywords:** Artificial intelligence, ChatGPT, Machine learning, Distance mapping, Statistical algorithm

## Abstract

Artificial intelligence (AI) is looked upon nowadays as the potential major catalyst for the fourth industrial revolution. In the last decade, AI use in Orthopaedics increased approximately tenfold. Artificial intelligence helps with tracking activities, evaluating diagnostic images, predicting injury risk, and several other uses. Chat Generated Pre-trained Transformer (ChatGPT), which is an AI-chatbot, represents an extremely controversial topic in the academic community. The aim of this review article is to simplify the concept of AI and study the extent of AI use in Orthopaedics and sports medicine literature. Additionally, the article will also evaluate the role of ChatGPT in scientific research and publications.

**Level of evidence**: Level V, letter to review.

## Introduction

The term artificial intelligence (AI) was first described by McCarthy et al. in 1955, when they called AI "the science and engineering of making intelligent machines". They thought that these machines would be able to do tasks that people used to think only humans could do, like abstract thinking and advanced problem solving [36]. Artificial intelligence also refers to the scientific and technological endeavor of developing intelligent computers that can perform functions normally associated with human effort [21]. There is a subset of AI known as machine learning (ML), which uses computational techniques to examine massive data sets in order to categorize, forecast, or obtain valuable information without explicit instructions [21]. The terms AI and ML are frequently used interchangeably [34].

Artificial intelligence is looked upon nowadays as the potential major catalyst for the fourth industrial revolution after steam engines in the 1760s, electricity and the petroleum revolution in the 1870s, and computers in the 1970s [35, 49]. Artificial intelligence has the potential to play such a role and provide new avenues as well as explore new frontiers in health care research and practice. In a recent study, single electronic medical record (EMR) research identified over 30.000 unique data items per patient [47]. “Inadequate time, insufficient context, and insufficient presence" make it difficult for physicians and researchers to synthesize data and make therapeutic decisions in an era of information overload. AI's predictive powers might help with economic sustainability and data surfeit [47]. The aim of this review is to simplify the concept of AI as well as evaluate its application in Orthopaedics in general and sports medicine in particular. Additionally, the article will discuss the rising and controversial role of Chat Generated Pre-trained Transformer (ChatGPT) in academia.

## How does it work?

Artificial intelligence uses sophisticated statistical techniques to analyze and interpret complicated relationships between variables. These algorithms can "learn" from data with minimal human programming. It uses a huge dataset that is divided into predictors such as graft diameter or associated injuries and outcomes, for example, graft failure, non-union, or revision procedures. The computer model analyzes each set of "predictor" characteristics to predict a certain result. The study may discover key elements, measure and rank them, and design an algorithm to predict the result. These orthopaedic algorithms may be utilized for future patients [33]. In other words, given a set of patient specific-data, for example, radiologic imaging, lab results, or any other data from electronic medical records, a diagnosis could be made, a risk score could be evaluated, or some treatment options could be evaluated [11].

Several modalities are used to accomplish the process of getting a meaningful output from input data. In general, algorithms could be categorized as supervised (the algorithm is trained by comparing its outcome to correctly labeled outputs) or unsupervised (the algorithm autonomously searches for patterns without trial-and-error training). The following table summarizes these methods (Table 1).

**Table 1:** Summary of different artificial intelligence modalities

Method	Description
**1. Expert systems** [57]	Early AI systems that replicate "expert" decisions. These systems use knowledge bases with organized, factual deductions and heuristics. An expert system learns from these facts and creates "rules" for future decision-making
**2. Logistic** **regression** [45]	Predicts binary response variables using a logistic function. It provides simple and reproducible results to compare different complex models
**3. Bayesian** **networks** [38]	These models illustrate variable-outcome connections. They model outcome probability distributions as local, conditional discrete variable probability distributions. It might predict injury risk for an athlete based on current performance measurements and injury history
**4. Random Forest algorithms** [45]	These algorithms build many "decision trees," flowchart-like structures that emerge from decisions at numerous branching decision points
**5. Support vector machines** [45]	It creates a multidimensional representation of data as points in space, mapped to distinguish categories as clearly as feasible
**6. Artificial and deep neural networks** [18]	These models are more independent and require little to no human supervision with less data reformatting

### Chat Generative Pre-trained Transformer (ChatGPT)

It is the newest member of the AI family and has found its way very rapidly into healthcare services and research. ChatGPT uses a hybrid type of language formatting that includes supervised learning as well as non-supervised or reinforcement learning with human feedback (RLHF). It simply generates an output report depending on the inputs provided. It has the potential advantage of providing an overview of the existing literature about a certain topic, detecting some existing knowledge gaps, and providing novel ideas or hypotheses for research [13]. Searching PubMed on June 6th, 2023, for the term "ChatGPT" revealed 564 articles (560 published in 2023). This chatbot had even been tested to pass high-level exams such as the United States Medical License Exam (USMLE) and the American Board of Orthopaedic Surgery (ABOS) exam [31, 33]. No one doubts the high potential and capabilities of different AI tools such as ChatGPT; however, there are several concerns about their application in health care services and research (which will be discussed later in a separate section).

## Current status of artificial intelligence use in orthopaedics

In a systematic review published in 2018, Cabitza et al. showed a trend of increased use of AI in Orthopaedics with an almost tenfold increase since 2010 [11]. They also found that AI was mainly used for diagnostic purposes, for example, osteoarthritis prediction or detection, joints, bones, and spine pathology imaging. The following table provides insight about the use of AI in Orthopaedics (Table 2).

**Table 2:** Different examples of artificial intelligence (AI) use across several orthopaedic sub-specialties

Scope	Examples
**1. Fractures detection and prediction** [41]	- Evaluate the accuracy of deep neural networks to diagnose neck femur fractures in comparison to perceptual training of medically naïve individuals [1]. - Predict hip fractures and estimate predictor importance in Dual-energy X-ray absorptiometry (DXA)-scanned individuals [30] - Evaluate the ability of convolutional neural network to detect distal radius fracture on an antero-posterior view of the wrist [19]. - Incorporate diverse measurements of bone density and geometry from central QCT imaging and of bone microstructure from high-resolution peripheral QCT imaging, can improve fracture prediction [6].
**2. Osteoarthritis and arthroplasty**	- Compare different gait patterns in patients with uni-compartment knee arthroplasty versus total knee arthroplasty [22]. - Early prediction of symptomatic knee osteoarthritis using MRI images [5, 43]. - Develop machine-learning based implant recognition system for hip arthroplasty designs [24]. - Measures of knee cartilage thickness can predict future loss of knee cartilage [23]. - Investigate the quantification of osteoarthritis and prediction of tibial cartilage loss by analysis of the tibia trabecular bone from magnetic resonance images of knees [32]. - Knee cartilage segmentation using a tri-planar convolutional network [44]. - ML tool demonstrates clinical utility with early prediction of patients who are most at risk of developing poor postoperative functional outcomes and PROMs after primary total knee arthroplasty [10]. - Predict length of stay, discharge disposition, and inpatient charges for primary anatomic, reverse, and hemishoulder arthroplasty [26].
**3. Spine surgery**	- Classification of scoliosis curves [2]. - Detection of lumbar spine compression fractures [3]. - Using a handgrip device and target tracking test to detect impairments of hand motor function in patients with cervical spondylotic myelopathy [31]. - Detection of spinal metastasis using a multi-resolution approach [56].
**4. Foot and Ankle surgery**	- Using automated segmentation to study distance and coverage mapping in Chopart joints in patients with progressive collapsing foot deformity (PCFD) [7, 8]. - Advanced semi-automated segmentation to evaluate hallux rigidus [15]. - Objective Computational technique to classify ankle osteoarthritis on weight bearing computation tomography (WBCT) [51]. - Semi-automated assessment of different hallux valgus parameters on (WBCT) of the hallux valgus [16]
**5. Miscellaneous**	- Switching neural networks used to classify multiple osteochondromas [37]. - Develop a machine learning algorithm to predict the prolonged opioid use after total hip arthroplasty (THA) [25]. - Online image messaging platform for remote monitoring of surgical incision sites [58]. - Ensemble learning techniques to study skeletal maturity [12]. - Classify pathological gait patterns using 3D ground reaction force (GRFs) data [4].

## Artificial intelligence and sports medicine

Nowadays, there is widespread use of several smart tracking devices and phones, which are not only used by professional players but also amateur athletes and regular individuals during their daily life activities. The amount of data gathered by these devices and the development of deep learning and machine learning modules may increase the usefulness of these tracking devices. We could expect individually tailored treatment plans of care from a special training protocol to mitigate the risk of certain injuries and expect to return to play after sustaining sports injuries [45]. Artificial intelligence is becoming an integral pillar in modern sports medicine practice. Since professional sports across the world are a multibillion-dollar enterprise, optimizing players health status by decreasing injury risk has become a very crucial part of today’s sport. Karnuta et al. used an advanced ML algorithm to predict the next-season injury in hockey players with an accuracy of 94.6% (SD 0.5%) with good to excellent dependability [27]. Likewise, AI has been widely studied for image interpretation in radiology as well as other orthopaedic disciplines, and it is now slowly making its way into sports medicine practice and research [3, 19, 56]. Štajduhar et al. used a semi-automated technique to evaluate magnetic resonance imaging (MRI) images to detect anterior cruciate ligament (ACL) injuries. The area under the curve for complete rupture detection was 0.94 (which indicates excellent diagnostic accuracy) [50]. Kottie et al. were able to detect knee injuries from gait analysis using several parameters of ground reaction forces such as slope, direction, and push-off time [29]. Artificial intelligence was also used in sports medicine to predict possible changes in patient reported outcomes (PROs) after a procedure. Nwachukwu et al. used a specific ML algorithm to identify salient predictive variables that led to a clinically significant difference across three different hip scores in patients with femoro-acetabular impingement (FAI) [40]. The 3D distance mapping (which assesses the relative position between two opposing articular surfaces), coverage mapping (which utilizes the calculated distance maps to provide insights about areas of abnormal coverage), and volume measurements (which calculate the 3D volume amount of certain areas on WBCT images) of ankle syndesmosis have been recently studied in patients with progressive collapsing foot deformity (PCFD) [39]. It is possible that these new automated and semi-automated measurements will help untangle the confusion about the diagnosis of syndesmotic instability.

## Advanced distance mapping algorithm in orthopaedics

**Fig. 1:**
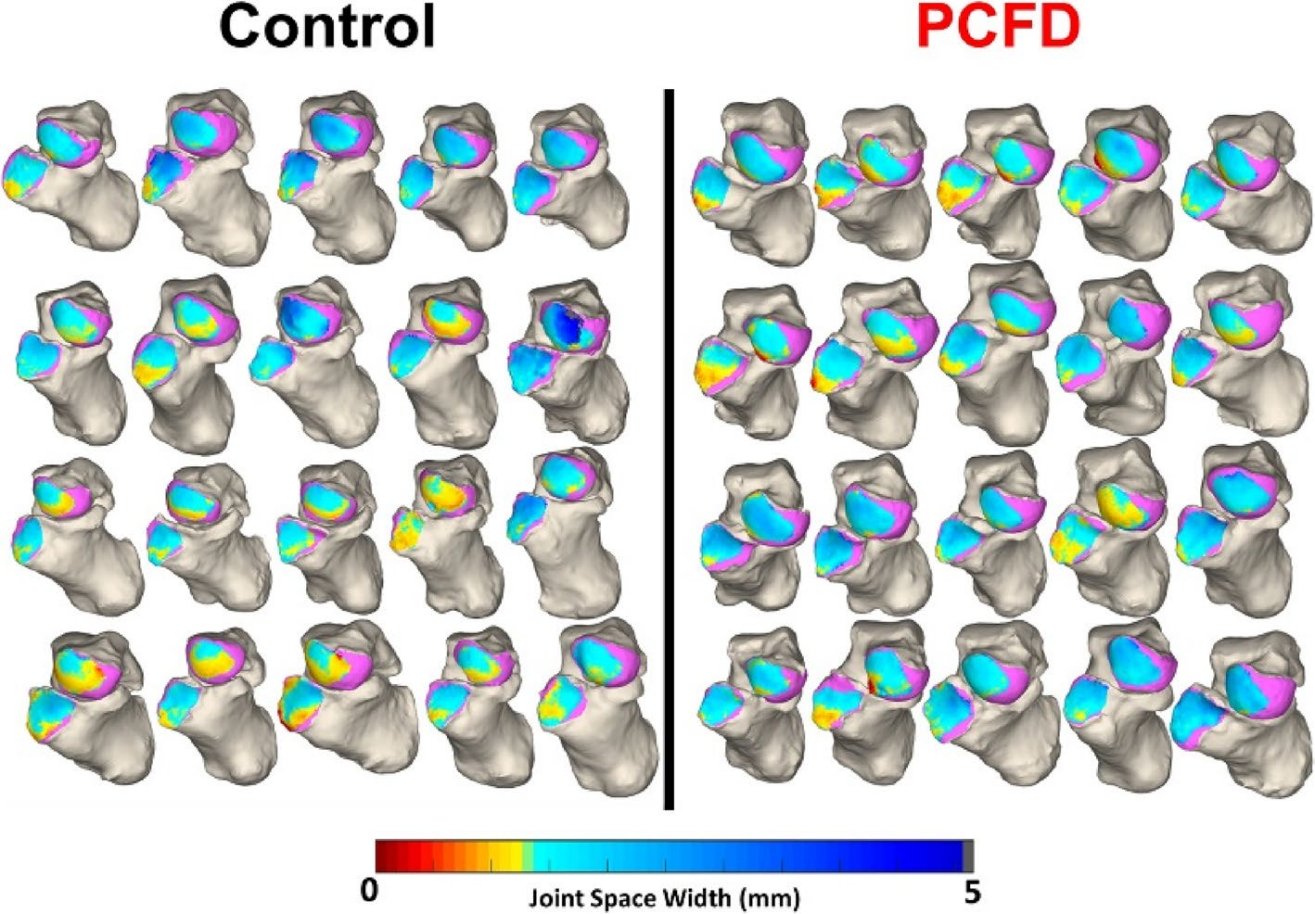
3D distance mapping in patients with progressive collapsing foot deformity (PCFD) versus control group

**Fig. 2:**
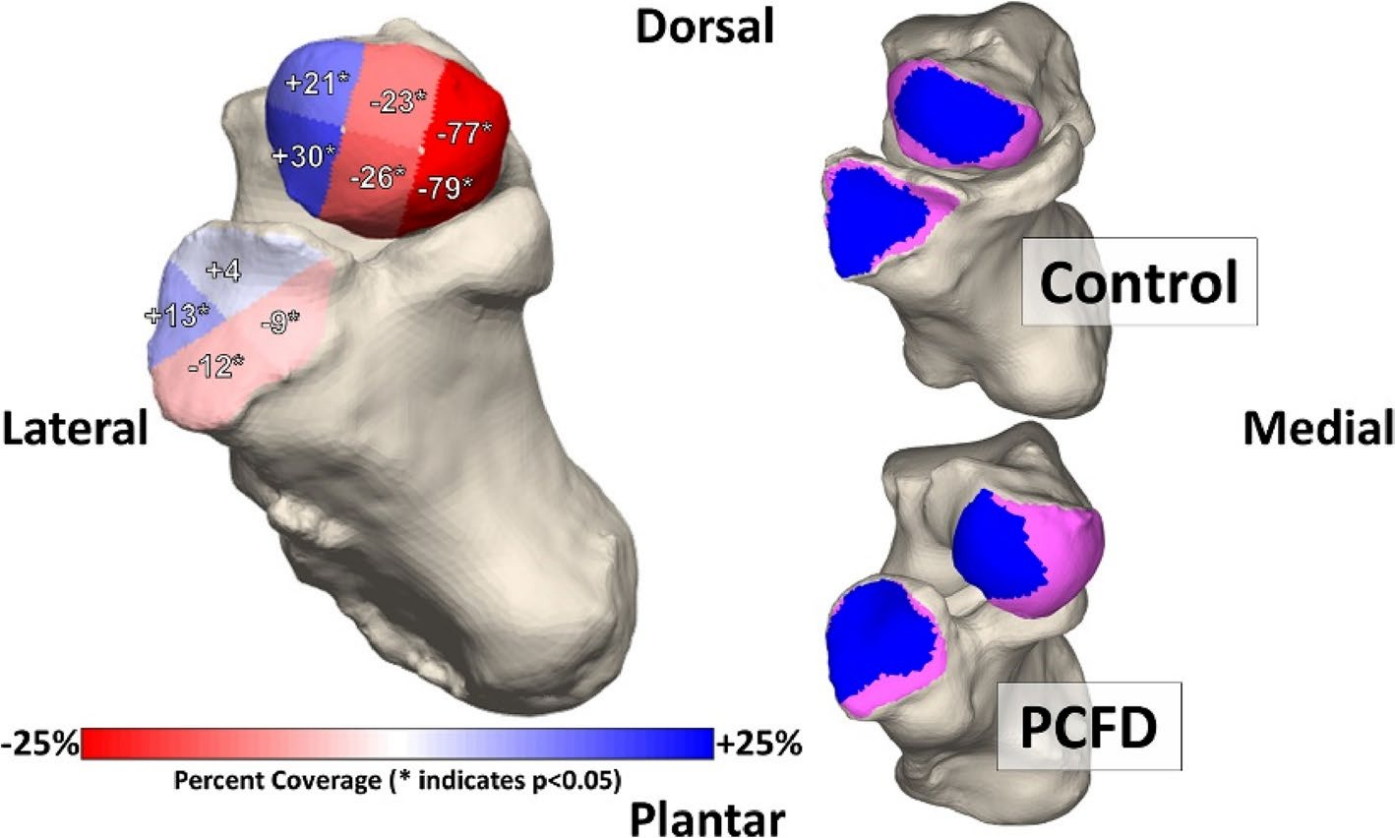
3D distance mapping in patients with progressive collapsing foot deformity (PCFD) versus control group

**Fig. 3:**
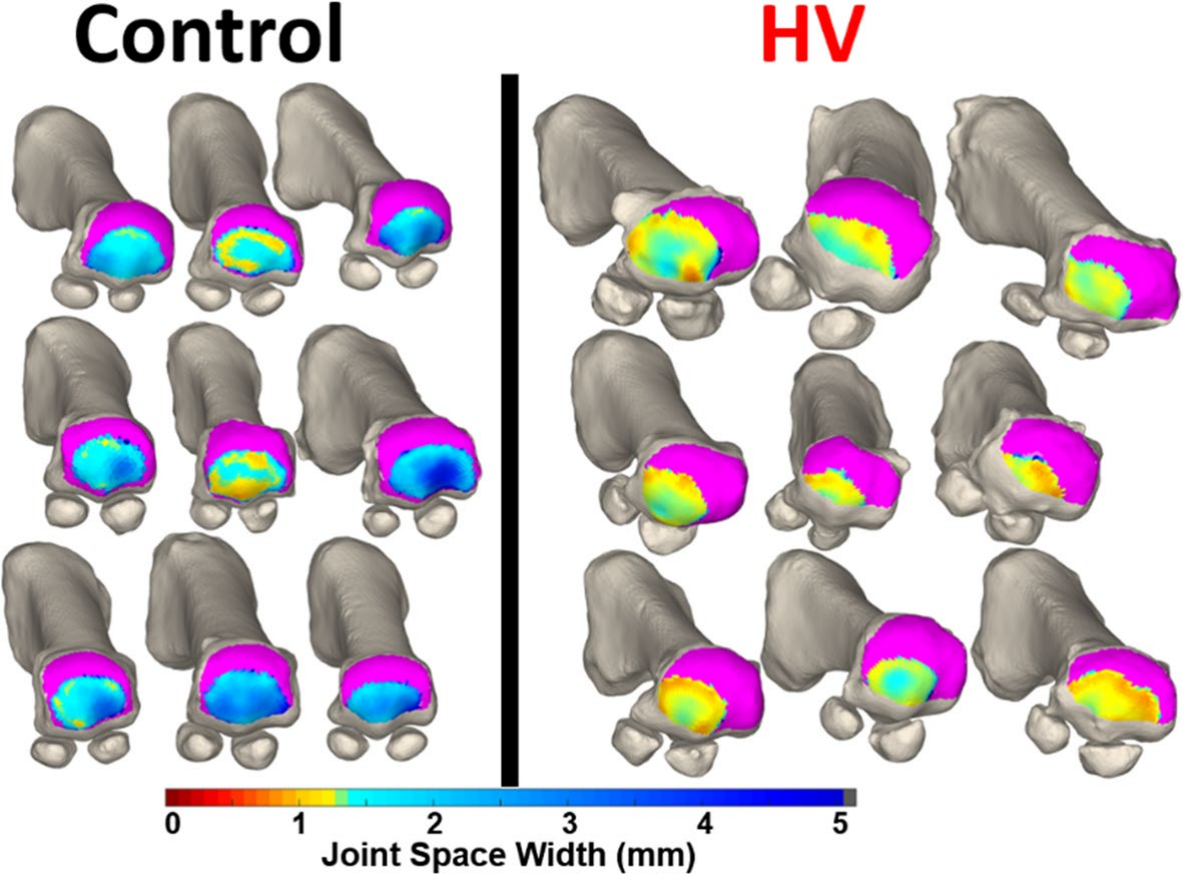
Distance mapping of the first metatarsophalangeal joint in patients with hallux valgus (HV) in comparison to a control group

**Fig. 4:**
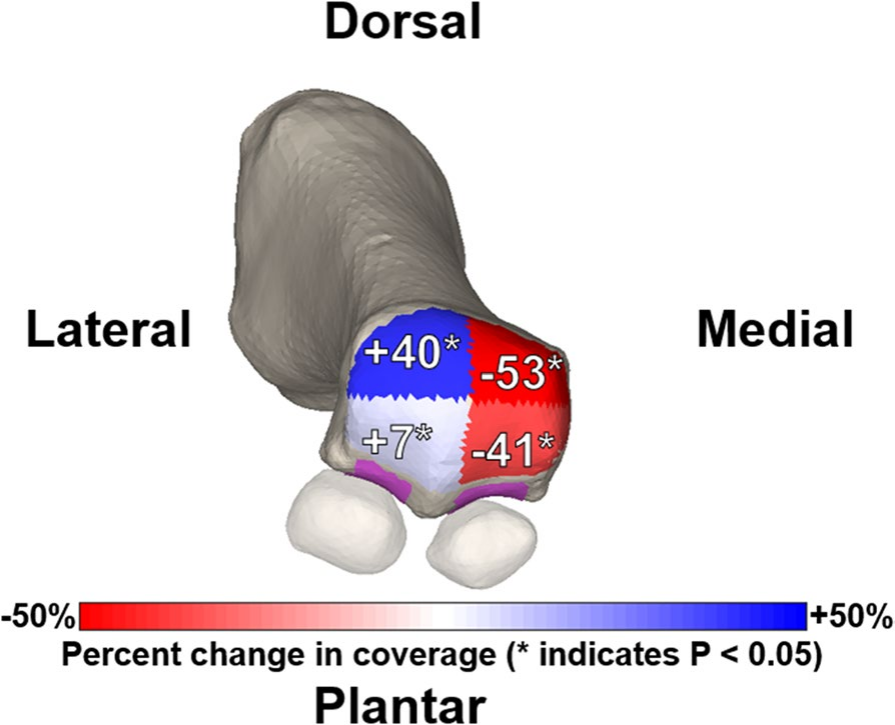
Distance mapping of the first metatarsophalangeal joint in patients with hallux valgus (HV)

Weight-bearing computed tomography (WBCT) has recently been used to assess a variety of lower extremity deformities and pathologies, such as knee osteoarthritis [46], ankle arthritis, progressive collapsing foot deformity [17], and hallux valgus [14] WBCT more accurately measures bone positioning than traditional weight-bearing radiographs and non-weight-bearing CT [48]. Previous studies have focused on using two-dimensional (2D) radiographs with manually calculated distances across a joint. However, recent studies have begun to shift to a more comprehensive approach, mapping the joint space width in three dimensions across the entire articulation of interest. These novel, three-dimensional methods provide superior characterization of the joint, made possible by automated joint mapping and ML-informed segmentation techniques. Examples in the literature include 3D mapping of the Chopart joint in patients with PCFD [7], the results of which are illustrated in Figs. 1 and 2. Another example is the use of distance mapping to characterize changes in the first metatarsophalangeal joint in patients with hallux valgus (bunion), as illustrated in Figs. 3 and 4.

### Navigating concerns and potential solutions in AI integration in healthcare

After reviewing what AI is capable of across different fields, health care professionals can start tailoring this new technology to best serve their scope of practice. Although we are living in an era of exponential growth in AI use, this technology comes with several concerns that must be tackled very well to achieve the best possible outcomes. First, there are concerns about a decrease or break in the physician–patient relationship with the increased use of technology in modern medical practice. Actually, AI could be a very useful tool to strengthen the physician–patient relationship by decreasing the time physicians spend navigating electronic medical records. Artificial intelligence could present patient-specific data in a very organized and stratified way that even makes the physician very aware of all the fine details of his patients, which will help build a stronger rapport with their patients. Second, the AI "black box phenomenon" is a source of concern to several physicians as the development of outcomes from different algorithms can’t be tracked, which could render certain outcomes unquestionable (especially in deep learning modules) [53]. There are also concerns about conflicts of decisions or potentially wrong AI outcomes (especially in the early use of this technology), which could decrease confidence levels at the physician or patient level or deskill physicians and turn them into machine-dependable. However, with judicious and supervised introduction and use of AI in practice, in addition to continuous appraisal and development of algorithms, we believe that the accuracy and precision of AI will get better over time.

### The impact and imperative of regulating ChatGPT in scientific publishing

Artificial intelligence, especially in its very recent form, ChatGPT, plays a very controversial role in the scientific and academic community. It was even listed as the author of several peer-reviewed, indexed articles [28, 54]. ChatGPT was also capable of writing abstracts and manuscripts that were difficult to distinguish from human abstracts, even by experts in the field [55]. However, the AI-generated articles carry a high risk of bias, inaccuracy, and misleading data [42]. In a study by Bhattacharyya et al., they found that ChatGPT-generated articles had only 7% authentic references, while the rest were either fabricated or authentic but inaccurate [9]. Since scholarly articles are the gatekeepers for the current body of scientific evidence and future directions, it becomes necessary to set rules and regulations for this double-edged sword. In a proactive move from the scientific community, authors now should sign a license not only to indicate that their work is original but also to explicitly prohibit the use of AI-generated materials such as texts, figures, and images [52]. The academic community also needs to cooperate with AI developers to validate programs to detect AI-generated articles, as is the case in plagiarism checking [20]. Training ChatGPT processing to be limited only to peer-reviewed articles or at least prioritized over other non-peer-reviewed articles could help increase the quality of its output. Moreover, AI could be used with caution as a research assistant to help summarize an article, generate potential research questions, extract relevant data such as authors or dates of publications, etc. Until more discrete regulations of AI rule in the academic world, the whole scientific community should judiciously use it with integrity, honesty, and transparency.

## Conclusion

To keep up with the ever-increasing sophistication of artificial intelligence, orthopaedic surgeons must be familiar with and able to implement a variety of AI-based approaches and modalities. Without a doubt, the field of orthopaedic surgery has a wealth of human and material resources that may be used to advance artificial intelligence and harness it to serve patients optimally.
